# City Planning and Governing of the communities 

**Published:** 2019-08

**Authors:** Guldbrand Skjönberg

**Affiliations:** ^*a*^International Safe Community Certifying Centre, Sweden.

## Abstract

A community has many different opportunities to improve the traffic safety. The community is local part of the public sector. On all decision made has the community big impacts on questions within its own territory.

Urban planning is an important tool. Urban planning is normally a rather complex system and important decisions are made in a long term perspective. It is very important for all who work for traffic safety to learn the system in order to have an impact.

The big pallet a community has embrace also many other tools than urban planning.

1. Reduce the need of transportation: Urban planning, public transportation, road toll, forbid use of cars in some areas, one-way roads, reducing parking places

2. Separation of areas for activities: Special lines for pedestrians, bikes, cars and public transportation

3. Safety in the road system: Roundabout, fens, avoid U-turns, street lightning, traffic light, sleeping policemen

4. Maintaining of the infrastructure: No digging technics, cleaning streets from snow fist for pedestrians

5. Supervision: Speed control, immediate feed-back to offenders. Camera supervision, Control of parking, Alco control, Control the use of helmets, belts etc., Control of vehicles

6. Local regulations: Speed limits, one way roads, parking regulations, no access for cars and motorbikes to some areas, the high of hedges, restrictions for traffic during some time during the day

7. Education and Information: Special parks for education of children, education and information in preschools and schools, education for old citizens

8. Support to different groups: Baby and child seats for rent, helmets for rent, reflectors, bus-service for elderly

9. Volunteers: Follow children to and from school, give advice to all in traffic, help the police in their supervision

10. Support to local sport-clubs and other associations

11. Restaurants and alcohol beverages: Restrictions to serve drivers alcohol, education to serving personnel, home driving service

12. Agreements with employees and tenders: Use of safety helmets, belts etc., intelligent speed adaptation (ISA), always give priority to public transportation or bike, use only safe cars with alcohol-interlock

**Keywords:**

City Planning, Governing, Safe Community

